# Mitochondrial genomic integrity and the nuclear epigenome in health and disease

**DOI:** 10.3389/fendo.2022.1059085

**Published:** 2022-11-07

**Authors:** Amanda L. Morin, Phyo W. Win, Angela Z. Lin, Christina A. Castellani

**Affiliations:** ^1^ Department of Pathology and Laboratory Medicine, Schulich School of Medicine and Dentistry, Western University, London, ON, Canada; ^2^ Department of Epidemiology and Biostatistics, Schulich School of Medicine and Dentistry, Western University, London, ON, Canada

**Keywords:** mitochondrial DNA, epigenome, disease, aging, metabolism, DNA methylation, histone acetylation

## Abstract

Bidirectional crosstalk between the nuclear and mitochondrial genomes is essential for proper cell functioning. Mitochondrial DNA copy number (mtDNA-CN) and heteroplasmy influence mitochondrial function, which can influence the nuclear genome and contribute to health and disease.

Evidence shows that mtDNA-CN and heteroplasmic variation are associated with aging, complex disease, and all-cause mortality. Further, the nuclear epigenome may mediate the effects of mtDNA variation on disease. In this way, mitochondria act as an environmental biosensor translating vital information about the state of the cell to the nuclear genome.

Cellular communication between mtDNA variation and the nuclear epigenome can be achieved by modification of metabolites and intermediates of the citric acid cycle and oxidative phosphorylation. These essential molecules (e.g. ATP, acetyl-CoA, ɑ-ketoglutarate and S-adenosylmethionine) act as substrates and cofactors for enzymes involved in epigenetic modifications.

The role of mitochondria as an environmental biosensor is emerging as a critical modifier of disease states. Uncovering the mechanisms of these dynamics in disease processes is expected to lead to earlier and improved treatment for a variety of diseases. However, the influence of mtDNA-CN and heteroplasmy variation on mitochondrially-derived epigenome-modifying metabolites and intermediates is poorly understood. This perspective will focus on the relationship between mtDNA-CN, heteroplasmy, and epigenome modifying cofactors and substrates, and the influence of their dynamics on the nuclear epigenome in health and disease.

## Introduction

The mitochondrion is a membrane bound organelle that plays a role in several cellular processes, including cellular metabolism, reactive oxygen species (ROS) production, and apoptosis (1). A crucial function of the mitochondrion is the generation of ATP through the tricarboxylic acid (TCA) cycle and oxidative phosphorylation (OXPHOS). Mitochondria contain circular genomes (mitochondrial DNA; mtDNA) that are maternally inherited, haploid, non-intronic, and ~16kb in length. mtDNA codes for 13 proteins that help compile 4/5 OXPHOS enzymatic complexes, and the tRNAs and rRNAs required for mitochondrial protein translation. Mitochondrial gene expression varies across different cells and tissues (2). Integrity of mtDNA is essential for energy production and overall mitochondrial function.

Two metrics of mtDNA integrity are mtDNA copy number (mtDNA-CN) and heteroplasmy. mtDNA-CN refers to the number of mitochondrial genomes present in a cell. Basal mtDNA-CN is cell- and tissue-specific, based on the energy demands of that cell/tissue; for example, cardiac tissue has a higher basal mtDNA-CN than lung epithelial tissue (3). mtDNA-CN is associated with health status (4), decreases with age (5, 6) and is higher in females (7). Heteroplasmy refers to the presence of genotypically diverse mtDNA molecules within a cell and increases with age (5, 6). Heteroplasmic burden is the ratio of mutated to wild-type mtDNA that determines a mutation’s likelihood to be phenotypically detrimental. When a threshold of heteroplasmic burden is exceeded, mitochondrial diseases may manifest (8). The evolution of mitochondrial genomes can be tracked by sequencing and grouping mtDNA molecules with similar genomic characteristics into groups called haplotypes. mtDNA-CN, heteroplasmy and mtDNA haplotypes have direct effects on the health and functioning of mitochondria which affects cell functioning (9). mtDNA variation and mitochondrial function are associated with a variety of diseases (10), as well as aging, frailty, and all-cause mortality (7). Generally, mtDNA-CN decrease and increased heteroplasmy are associated with disease (11, 12).

The mechanisms of the association between mtDNA variation and the epigenome have not been fully elucidated. The role mtDNA plays in epigenome dynamics was first revealed in 2008 in a cell model of ethidium bromide (EtBr)-mediated mtDNA depletion where differentially methylated nuclear DNA (nDNA) was observed (13). Since then, several models of mtDNA variation have been utilized to ascertain the mechanisms through which mtDNA influences the epigenome (9) (14–19). This link between mtDNA and the epigenome represents a promising avenue for further research as it may prove to be a useful predictor of disease.

**Figure 1:**
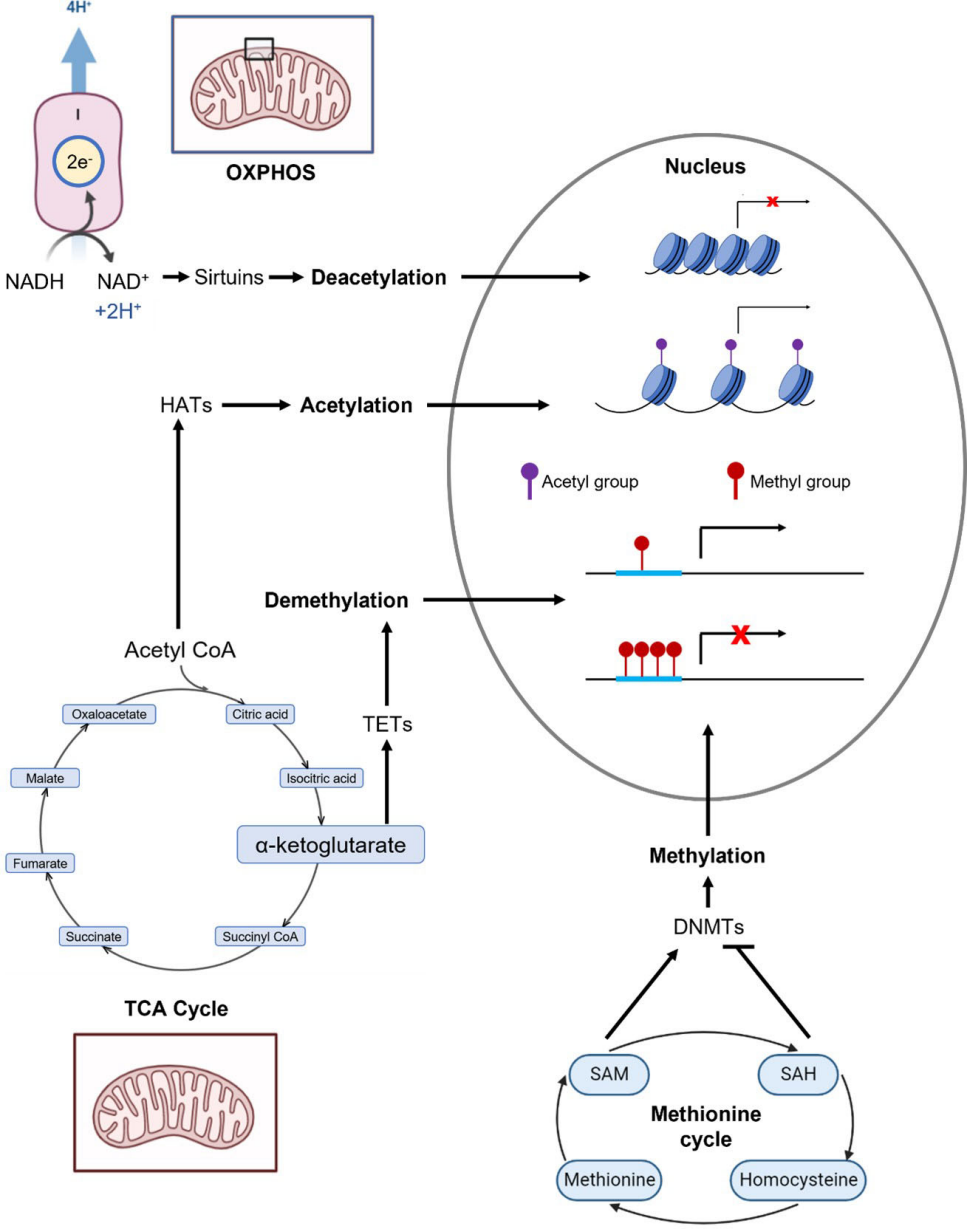
Overview of metabolic pathways that generate selected epigenome-modifying metabolites: methionine, S-adenosylmethionine (SAM), S-adenosylhomocysteine (SAH), a-ketoglutarate (aKG), acetyl CoA, and NAD+. The enzymatic reactions and epigenomic modifications of these metabolites are summarized in **Table 1**. Complex I of OXPHOS oxidizes NADH to NAD+. Acetyl CoA and aKG are metabolites of the TCA cycle. Methionine, SAM and SAH are critical components of the methionine cycle.

We and others propose that mtDNA influences the epigenome through cellular metabolism and modification of epigenome-modifying metabolites of the TCA cycle and OXPHOS. Some major known epigenome-modifying metabolites from the TCA cycle and OXPHOS are methionine, S-adenosylmethionine (SAM, promotes methylation), S-adenosylhomocysteine (SAH, inhibits methylation), alpha-ketoglutarate (aKG, promotes DNA demethylation), acetyl CoA (promotes histone acetylation), and NAD+ (promotes histone deacetylation). An overview of how these metabolites interact with the epigenome is presented in **Figure 1**; a summary of these metabolites and their interactions is presented in **Table 1**.

**Table 1:** Summary of selected metabolites, enzymatic interactions, and related epigenomic modification upon increase of the metabolite.

**Metabolite**	**Enzymatic Interaction**	**Epigenomic modification**
Methionine	Substrate for MAT, required for SAM production	Promotes DNA methylation
SAM	Substrate for DNMTs; activates activity	Promotes DNA methylation
SAH	Substrate for DNMTs; inhibits activity	Inhibits DNA methylation
α-ketoglutarate	Co-factor for TET and JMJD demethylases	Promotes DNA demethylation
Acetyl CoA	Substrate for HATs	Promotes histone acetylation
NAD+	Co-factor for sirtuins	Promotes histone deacetylation

We hypothesize that modification of the TCA cycle and OXPHOS through mtDNA-CN and heteroplasmy variation modulates the availability of epigenome-modifying metabolites, influencing the epigenome and gene expression in regions associated with complex disease. In this perspective, we will briefly review the importance of mitochondrial-nuclear cross-talk in maintaining genomic integrity and cell function, the effects of mtDNA variation on mitochondrial function, and the association between mtDNA, aging and disease. We further review the known connections between mtDNA, epigenomic changes, and differential gene expression. Finally, we summarize the proposed role for a subset of epigenome-modifying substrates and co-factors required for methylation/demethylation and acetylation/deacetylation in nuclear epigenomic reactions.

## Mito-nuclear cross-talk is essential for proper cell functioning

Replication and transcription of mtDNA is controlled by nuclear-encoded genes, therefore nDNA plays a pivotal role in maintaining mitochondrial function and genomic integrity. mtDNA polymerase gamma (*POLG*), the catalytic subunit of mtDNA polymerase, is the primary polymerase responsible for mtDNA replication. With a polymerase and exonuclease domain, *POLG* synthesizes and edits new mtDNA strands (17). Mitochondrial transcription factor A (*TFAM*) also plays a role in mtDNA replication and transcription (14). nDNA encodes all but 13 protein subunits required for energy metabolism and maintenance of mitochondrial function. It is important that these genomes communicate *via* anterograde (nucleus to mitochondria) and retrograde (mitochondria to nucleus) signaling to maintain genomic integrity and function.

An example of retrograde signaling in genome integrity and cell function is during the cell cycle. nDNA-encoded cell-cycle checkpoint proteins such as p21 are upregulated in response to double stranded breaks in mtDNA (20). During the S-phase of the cell cycle, mitochondrial activities ensure the translocation of mitochondrial enzymes that alter nuclear epigenetic marks to make cell cycle-related genes more accessible during DNA replication (21). Another example involves mitochondrial nuclear retrograde regulator 1 (*MNRR1*), a bi-organellar protein that activates respiration by binding to cytochrome c oxidase in mitochondria and acts as a transcriptional activator in the nucleus by binding to a conserved oxygen-responsive promoter element of several stress-related genes (22). Another example of mito-nuclear cross-talk is in embryogenesis, initiated by maternal cytoplasmic factors until zygotic genome activation (ZGA) occurs (23). Mitochondria contribute to metabolite production required for ZGA and generates signals needed to transport these metabolites and other enzymes to the nucleus (24). Mito-nuclear cross-talk is essential for other developmental activities, such as cell specification and differentiation (25).

It is clear that mtDNA and nDNA work together to maintain the needs of our cells. This essential relationship can be further assessed to elucidate the mechanisms that link mtDNA variation with the epigenome.

## Mitochondrial DNA variation and mitochondrial function

Variation in mtDNA-CN or heteroplasmy affects mitochondrial function. Generally, decreasing mtDNA-CN and/or increasing heteroplasmy negatively affects mitochondrial function; conversely, increasing mtDNA-CN and/or decreasing heteroplasmy enhances mitochondrial function (26).

mtDNA-CN and heteroplasmy vary in response to a variety of environmental factors. Some lifestyle factors such as obesity have been shown to decrease mtDNA-CN (27), while alcohol consumption and cigarette smoking can lead to mtDNA deletions (28, 29). Many environmental pollutants such as heavy metals and polyaromatic hydrocarbons can increase mtDNA-CN (30, 31). Life-saving pharmaceuticals such as the HIV antiretroviral drug Zidovudine significantly decrease mtDNA-CN (32), and stressful life events have been suggested to modify mtDNA (33).

Several cell models of mtDNA variation exist. *POLG* mutated in the exonuclease or polymerase domain modifies heteroplasmy and mtDNA-CN, respectively. Mutated *POLG* exhibits a dominant-negative phenotype, wherein the activity of mutated POLG inhibits activity of wild-type POLG; polymerase domain mutations can reduce mtDNA-CN up to 50% with every cell division, while exonuclease domain mutations can produce 5-10-fold mutational loads in mtDNA (18). Further, dominant-negative *POLG* (DN-*POLG*) expression can be induced, allowing fine-tuned control of mtDNA-CN and heteroplasmy variation, making this a useful system to assess the effects of mtDNA variation (17–19). *TFAM* can be mutated or knocked out/down to alter mtDNA-CN. CRISPR-mediated knockout of *TFAM* results in an 18-fold decrease in mtDNA-CN (14); RNAi-mediated knockdown of *TFAM* with siRNA (si-*TFAM*) decreases mtDNA-CN by ~40% (16).

Modification of heteroplasmy leads to modified mitochondrial function. Cells with 73% heteroplasmy exhibit defective mitochondrial function and low expression levels of mtDNA-encoded proteins (22). In a separate cell line, increased heteroplasmy is associated with mitochondrial transcript reduction, and causes a dose-dependent reduction in mtDNA-encoded protein expression (34). In a study analyzing nine European haplotypes, cells carrying mtDNA haplotype J have lower levels of intracellular ATP and ROS, indicating decreased OXPHOS efficiency (35).

Increased mtDNA-CN is associated with an increase in mitochondrial gene expression and correlates with an increase in mitochondrial function (36). This rise in mtDNA-CN is regularly seen in embryogenesis and differentiation (15). Natural variation of mtDNA-CN is seen across tissues from different individuals and can influence mitochondrial function; for example, human skeletal muscle samples with a higher mtDNA-CN display increased activity of mtDNA-encoded OXPHOS complex proteins (37). In general, increased mtDNA-CN increases mitochondrial function.

Decreased mtDNA-CN is associated with decreased expression of mtDNA-encoded OXPHOS complex subunits, inhibition of complex I, III, IV and V activity, and limited ATP production (13). Disruption of gene expression and inhibition of complex activity compromises cellular respiratory capacity (38). Cells with low mtDNA-CN show partial OXPHOS defects and prioritize glutamine metabolism for chemical energy production (39). Furthermore, inhibiting complex I activity increases superoxide production (40). OXPHOS replenishes NAD+ pools for the TCA cycle; inhibition of OXPHOS complex activity *via* mtDNA decrease perturbs TCA cycle activity, altering the metabolic state of the cell (19).

## The mitochondria in aging and complex disease

Mitochondrial dysfunction is implicated in several human diseases, including cancer (41), diabetes (42), cardiovascular disease (CVD) (43), HIV/AIDS (44), multiple sclerosis (45), Alzheimer’s, Parkinson’s, Huntington’s (34), autism (46), and schizophrenia (47). Mitochondria are also implicated as drivers of aging phenotypes (1, 20). There is a clear association that exists between mitochondrial function and disease, and mitochondrial function and aging.

Pathway analysis of cells with 50-90% heteroplasmy revealed an upregulation of aging and senescence pathways (34). Further, mice expressing exonuclease-deficient DN-*Polg* showed premature aging phenotypes such as kyphosis and hair loss as well as reduced lifespan (1), and mice treated with rotenone (complex I inhibitor) in early life exhibited a unique aging transcriptional profile (40). This suggests that increasing heteroplasmy and inducing mitochondrial dysfunction promotes the expression of gene pathways that give rise to aging phenotypes.

Mitochondria are heavily implicated in the pathogenesis of cancer (48). A hallmarks of cancer cells is the Warburg effect, wherein the cell metabolizes glucose primarily through glycolysis in the presence of oxygen. Often, nuclear-encoded mitochondrial genes are mutated in cancer, for example, isocitrate dehydrogenase (*IDH*), a key enzyme in the TCA cycle (49–51).

Cells of cardiac tissue have high basal mtDNA-CN, likely due to high energy demands; studies of mitochondrial contribution to cardiac-related diseases are plentiful. In a study of dilated cardiomyopathy (DCM) patients, lactate production was 5.4-fold higher in DCM patients than controls, and elevated aKG levels were seen (52). This may reflect a switch in metabolism towards the TCA cycle to compensate for decreased energy metabolism through anaerobic glycolysis. In a mouse model of heart failure, mtDNA-CN decreased by ~40% in failing myocardium after myocardial infarction (53). In another mouse model, symptoms of cardiomyopathy due to DN-*POLG* transgene expression were confirmed (54), and in another model, DN-*POLG* transgene expression led to left ventricle hypertrophy that progressed into cardiogenic heart failure (55).

Studies linking mitochondrial function to disease pathophysiology are too extensive for the scope of this perspective. However, evidence points to a clear association between mtDNA, mitochondrial function, aging, and disease. The epigenome and gene expression are implicated as major players in the association between mtDNA and disease which we will discuss below.

## Mitochondrial DNA variation is associated with epigenomic changes at specific nuclear loci and influences gene expression

Mitochondrial variation is associated with DNA and histone methylation changes. We performed an EWAS using methylation data from three CVD cohorts and identified CpGs significantly associated with mtDNA-CN; these CpGs were further validated in an *in vitro* model of mtDNA-CN depletion *via TFAM* knockout (14). Further, an EWAS and meta-analysis of mtDNA-CN association with DNA methylation (DNAm) revealed CpGs to be significantly associated with mtDNA-CN across multiple ethnicities in five cohorts (56). Results from a cybrid cell model suggest that variable histone methylation is highly dependent on mitochondria (57). mtDNA haplotype is a determinant of global DNAm levels (35). In a study analyzing murine embryonic stem cell (ESC) differentiation in response to different haplotypes, divergent haplotypes induced DNAm changes (58). In a mouse population with the same nDNA but different mtDNA, altering mtDNA leads to differential methylation (59). Culturing induced pluripotent stem cells (iPSCs) in 5% oxygen reduces H3K27 trimethylation which is restored when cultured in atmospheric 20% oxygen (60). In glioblastoma tumour cells with low mtDNA-CN, differential methylation is seen compared to cells containing 100% of their mtDNA (50). Furthermore, we determined that mtDNA-CN is causative of changes in nDNA methylation, evidenced by the fact that the methylated sites did not drive alterations in mtDNA-CN (14).

Mitochondrial variation is also associated with histone acetylation changes. Mitochondrial variation can lead to mitochondrial dysfunction, triggering mitochondrial superoxide production. Superoxide mediates the modification of several histone acetylation marks, including H3K9 and H3K14 (51). In cybrid cell models, mitochondria significantly contribute to H4K16 acetylation variation (57), and mitochondrial metabolism exerts some control over nuclear histone acetylation modifications (61). mtDNA-CN reduction leads to decreased HDAC activity, which increases histone H3K27 acetylation in gene promoters, likely triggering chromatin activation (15). mtDNA reduction also invokes a decrease in acetylation marks for H2B, H3 and H4 histones, though acetylation of these histones can be rescued upon TCA cycle restoration (19). Culturing iPSCs in 5% oxygen rather than atmospheric 20% oxygen reduces H3K27 acetylation which is restored when cultured in 20% oxygen (60). Taken together, the literature supports an association between mitochondrial variation and histone acetylation dynamics.

Mitochondrial variation and its influence on the epigenome is also associated with differential gene expression. DNAm profile analysis of ESCs from four mice strains with different mtDNA haplotypes reveal 8351 differentially methylated CpGs assigned to 4243 genomic loci, 3552 of which are known genes (58). Upon mtDNA-CN depletion, progressive increases of DNAm in promoters and gene bodies are seen; additionally, differential methylation occurs primarily in the promoter region of differentially expressed genes (DEGs) (62). In a mutant *IDH* cell line, differential methylation is seen in ~14 000 promoters (49). The human mtDNA J haplotype exhibits higher mRNA levels of the methyl adenosyl transferase (MAT) gene *MAT1A*, which replenishes SAM levels and could explain the haplotypes’ increased global DNAm (35). In a mouse model of mtDNA-CN depletion, 95 genes were differentially expressed (55), while a second mouse model of mtDNA-CN depletion reveals 121 DEGs (63).

These significant associations that exist between mtDNA variation and the state of the epigenome at specific genomic loci strongly indicate a direct relationship between mitochondrial function and the epigenome and transcriptome.

## Mitochondrial dysfunction alters metabolism and regulates epigenome modifying metabolites

The mechanisms of the relationship between mitochondrial function and the epigenome and transcriptome are yet to be elucidated. We propose that this relationship exists through mitochondrial metabolites that are known to be substrates and co-factors for epigenome-modifying processes. We have elected to review the metabolites outlined in **Table 1**: methionine (*MAT* substrate), SAM (donor for methylation), SAH (metabolite of SAM), aKG (co-factor for TET demethylases), acetyl CoA (substrate for acetylation) and NAD+ (cofactor for sirtuin deacetylases), as these metabolites are crucial to mitochondrial metabolism and are well-studied in their roles contributing to DNA and histone methylation and histone acetylation.

Studies of a well-known pharmaceutical, metformin, provide evidence that epigenomic changes could be mediated by mitochondrial function. Metformin significantly decreases SAH levels, thus increasing SAM levels, promoting DNAm (64). Metformin does not modify DNAm in cells depleted of their mitochondria, suggesting that metformin contributes to epigenomic changes *via* mitochondria (65).

The one-carbon cycle, also referred to as the folate cycle, includes reactions which occur both in the cytoplasm but also primarily in the mitochondria. This cycle reflects the transfer of one carbon from either serine or glycine generating methionine and/or key contributors to RNA and DNA. *Via* methionine, the one-carbon cycle contributes to the production of SAM. Thus, the one-carbon cycle can indirectly affect methylation through alteration of SAM, a methyl donor used in DNAm. These dynamics are evidenced by mtDNA-CN depletion triggering expression of key synthesis genes and enzymes of the one-carbon cycle and encouraging homocysteine remethylation (66, 67). Furthermore, mtDNA-CN depleted cells alter metabolism to produce serine from glucose (38). When glucose is metabolized to serine, intermediates of the TCA cycle and OXPHOS are modulated to compensate, for example decreasing pools of aKG, contributing to hypermethylation *via* decreased TET demethylase activity (63). The serine metabolism pathway fuels the methionine salvage pathway to help regenerate cellular levels of SAM (68), whose increase also contributes to hypermethylation (63). *MAT*s generate SAM from methionine; differential DNAm between haplotypes can be achieved through the modulation of *MAT* expression, which in turn modulates SAM availability for methylation reactions (35). Glucose metabolism can be altered by administration of 2-deoxyglucose; upon administration, global histone acetylation is altered, pointing towards glucose availability and mitochondrial function contributing to epigenomic changes (69).

The majority (70%) of acetyl CoA is derived from mitochondrially metabolized glucose; in cells with 100% heteroplasmy and increased mitochondrial dysfunction, glucose is metabolized to lactate, limiting production of acetyl CoA and inhibiting histone acetylation (61). mtDNA-CN depletion also results in diminished acetyl CoA pools, reduced HAT activity, and loss of histone acetylation peaks (62). The rest of cellular acetyl CoA is derived from other chemical sources, such as N-acetylaspartate (NAA). NAA is an amino acid derivative formed by the anabolism of aspartic acid and acetyl CoA. NAA is metabolized to aspartic acid and acetyl CoA *via* aspartoacylase (ASPA) activity and is a reaction that can replete both acetyl CoA and aspartic acid pools. When ASPA expression is knocked down, the abundance of acetyl CoA pools decreases (70), which likely stalls the TCA cycle, resulting in decreased histone acetylation.

*IDH* is an NAD+-dependent enzyme responsible for converting isocitrate into aKG and is a key enzyme of the TCA cycle. Altering or inhibiting *IDH* activity contributes to mitochondrial dysfunction, as is the case in many cancers where *IDH* mutations are present (49, 50, 71). Mutated *IDH* further metabolizes aKG to 2-hydroxyglutarate (2HG), which competes with aKG to inhibit the function of aKG-dependent enzymes, including TET demethylases, resulting in a significant increase in DNAm (49). 2HG dehydrogenases are evolutionarily conserved enzymes that metabolize 2HG back into aKG, likely mitigating the effects of mutant *IDH* (72). Mitochondrial superoxide, a primary by-product of oxidative stress, further contributes to mitochondrial dysfunction *via* inhibition of *IDH* activity resulting in accumulation of citrate and acetyl CoA and depleted aKG pools (51). This accumulation of acetyl CoA contributes to histone acetylation and transcriptional activation (60). Furthermore, this inhibition of isocitrate metabolism to aKG increases NAD+ pools since reduction of NAD+ to NADH happens concurrently to the oxidation of isocitrate.

We propose that mitochondria connect complex disease etiology to the environment and in this way act as a sensor of cell state. Non-Mendelian transmission and variable penetrance of complex diseases may in part be explained by the connection between mtDNA variation, mitochondrial function, cellular metabolism, and the nuclear epigenome (73). Although narrow, evidence in support of the role of environmental insults on mtDNA dynamics is increasing and suggests that endogenous and exogenous genotoxins mutate mtDNA yielding shifts in epigenome-modifying metabolites. For example, m.8993T>G results in mutated ATP synthase subunit 6 (*A6MT*) forcing cells to shift metabolic state to replenish aKG pools (39), while m.3243A>G reduces aKG and acetyl CoA pools (61). Thus, different mtDNA mutations arising from environmental impacts can contribute to unique epigenomic states, each with its own influence on the expression of underlying genes. In this way, mitochondria may act as an environmental biosensor, transmitting vital information about the state of a cell to the nucleus to modify gene expression.

## Conclusion

Mitochondria, as the energy generator of the cell, significantly contributes to overall cellular metabolism. The integrity of its genome and communication with nDNA is essential for proper mitochondrial function. mtDNA holds the information needed to translate 13 OXPHOS complex protein enzymatic subunits; as such, modulation of the quality (heteroplasmy) and quantity (mtDNA-CN) of the genome is essential for mitochondrial function. Modulation of mtDNA alters the translation of OXPHOS protein subunits, perturbing mitochondrial function.

Evidence suggests that mitochondrial function impacts the epigenome and transcriptome. Since the first reports of an association between mitochondria and the epigenome in 2008, many efforts have been put forward to elucidate this association. Using cellular and *in vivo* models of mtDNA-CN depletion and heteroplasmic burden, significant evidence exists for this association. We reviewed evidence that mtDNA variation and mitochondrial function contribute to epigenomic changes by modulating mitochondrial metabolites that act as substrates and cofactors for epigenomic processes. Given this significant evidence connecting mtDNA and mitochondrial function with cellular metabolism and the epigenome, rationale for follow-up studies exists, particularly for other epigenomic modifications (e.g., phosphorylation and propionylation) that are not studied as extensively as methylation and acetylation. Research into these associations could reveal new pathogenic pathways and allow for the research of treatments related to the pathophysiology of many diseases, particularly age-related complex disease.

## Data availability statement

The original contributions presented in the study are included in the article/supplementary material. Further inquiries can be directed to the corresponding author.

## Author contributions

ML conducted the literature search, created the figure, and wrote the bulk of the manuscript. WW and CC provided extensive edits and necessary literature. LZ edited for clarity and provided the content of the table. All authors contributed to the article and approved the submitted version.

## Funding

We thank the Department of Pathology and Laboratory Medicine at Western University and the Children’s Health Research Institute (CHRI), for support that led to this publication.

## Conflict of interest

The authors declare that the research was conducted in the absence of any commercial or financial relationships that could be construed as a potential conflict of interest.

## Publisher’s note

All claims expressed in this article are solely those of the authors and do not necessarily represent those of their affiliated organizations, or those of the publisher, the editors and the reviewers. Any product that may be evaluated in this article, or claim that may be made by its manufacturer, is not guaranteed or endorsed by the publisher.
